# Exosome-depleted MiR-148a-3p derived from Hepatic Stellate Cells Promotes Tumor Progression via ITGA5/PI3K/Akt Axis in Hepatocellular Carcinoma

**DOI:** 10.7150/ijbs.66184

**Published:** 2022-03-06

**Authors:** Xiangyu Zhang, Feiyu Chen, Peiran Huang, Xinyu Wang, Kaiqian Zhou, Cheng Zhou, Lei Yu, Yuanfei Peng, Jia Fan, Jian Zhou, Zuohua Lu, Jie Hu, Zheng Wang

**Affiliations:** 1 Department of Liver Surgery and Transplantation, Liver Cancer Institute, Zhongshan Hospital, Fudan University; Key Laboratory of Carcinogenesis and Cancer Invasion (Fudan University), Ministry of Education; Shanghai Key Laboratory of Organ Transplantation, Zhongshan Hospital, Fudan University, Shanghai, 200032, China.; 2 Institute of Biomedical Sciences, Fudan University, Shanghai, 200032, China.; 3 State Key Laboratory of Genetic Engineering, Fudan University, Shanghai, 200032, China.; 4 Department of Clinical Laboratory, Shanghai Pudong New Area Gongli Hospital, Shanghai, 200135, China.

**Keywords:** Exosome, MiR-148a-3p, Hepatic stellate cells, Hepatocellular carcinoma, Tumor microenvironment

## Abstract

Hepatocellular carcinoma (HCC) is a major cause of cancer-related death worldwide. Although it has been known that hepatic stellate cells (HSCs) play critical roles in the development and progression of HCC, the molecular mechanism underlying crosstalk between HSCs and cancer cells still remains unclear. Here, we investigated the interactions between HSCs and cancer cells through an indirect co-culture system. The expressions of cellular and exosomal miR-148a-3p were evaluated by quantitative real-time PCR. Cell counting kit-8 was used for evaluating cell growth *in vitro*. Cell migration and invasion ability were evaluated by wound-healing and Transwell assays. Western blot, quantitative real-time PCR and Luciferase reporter assay were performed to determine the target gene of miR-148a-3p. A xenograft liver cancer model was established to study the function of exosomal miR-148a-3p *in vivo*.

We found that miR-148a-3p was downregulated in co-cultured HSCs and overexpression of miR-148a-3p in HSCs impaired the proliferation and invasiveness of HCC both *in vitro* and *in vivo*. Moreover, further study showed that the miR-148a-3p was also downexpressed in HSCs-derived exosomes, and increased HSCs-derived exosomal miR-148a-3p suppressed HCC tumorigenesis through ITGA5/PI3K/Akt pathway. In conclusion, our study demonstrated that exosome-depleted miR-148a-3p derived from activated HSCs accelerates HCC progression through ITGA5/PI3K/Akt axis.

## Introduction

Hepatocellular carcinoma (HCC) is the most common primary liver cancer and the fifth leading cause of cancer-related death worldwide.^1^, ^2^ Chronic liver injuries, including hepatitis, alcohol consumption, and Non-alcoholic steatohepatitis (NASH), can induce activation of hepatic stellate cells (HSCs) and sequentially result in liver fibrosis and even progress to liver cirrhosis,^3^ which accounts for more than 90% of hepatocellular carcinoma.^4^ It is well known that the tumor microenvironment (TME), including cancer and stromal cells, is responsible for tumorigenesis.^5^

Hepatic stellate cells are the major source of cancer-associated fibroblasts (CAFs) in liver and play a vital role in fibrogenesis through extracellular matrix remodeling.^6^ They are critically involved in the tumorigenesis, tumor immunity and resistance to chemotherapy,^7^, ^8^ and recognized to be pleiotropic in regulating microenvironment with the secretion of various cytokines, displaying different capacities to influence the development of hepatocellular carcinoma.^9^, ^10^

Exosomes are membrane-bound nanosized extracellular vesicles of 50-100nm diameters secreted by various cells including tumor cells and contain a wide variety of bioactive substances, including proteins, lipids, and nucleic acids,^11^ which involve in multiple cell functions after the uptake of exosomes by target cells.^12^-^14^ Besides, tumor-derived exosomes are involved in mediating inflammation and inducing innate immune response,^15^ contributing to the vital role of inhibitory functions on tumor cells.^16^ Moreover, several studies indicated that exosomes could be utilized for drug delivery into tumor cells, avoiding the attenuation of drugs within the circulatory system and enhancing tumor-targeting by modifying the surface molecules.^13^, ^17^

Previous study has indicated that exosomal miRNAs can promote tumor development in a paracrine manner^18^ and contribute to the proliferation of cancer stem cells, chemoresistance, and enhanced malignancy of tumor.^19^ Recently, it has been shown that fibroblasts, the principal component of stromal cells, could be activated by dysregulated exosomal miRNAs in multiple type of cancers.^20^, ^21^ Moreover, exosomal miRNAs secreted by activated fibroblasts could also affected the phenotypes of tumor cells within the TME.^22^

In the present study, we employed a TME-like co-culture system of liver cancer cells and HSCs *in vitro*. Using co-culture systems and miRNA microarrays, we found the tumor-suppressor function of HSCs-derived exosomal miR-148a-3p in HCC through ITGA5/PI3K/Akt axis and highlighted the importance of TME in HCC development.

## Material and methods

### Patients and tissue samples

Primary HCC tumor and paired peritumor tissues were collected for isolating primary fibroblasts. Briefly, 17 primary HCC patient samples who meet the eligibility criteria (**Table S1**), were collected from Zhongshan hospital, Fudan University between December 2017 and February 2018. The freshly excised tissues were preserved in DMEM with 1% antibiotics and transferred at 4℃. All related procedures were approved by the Ethical Committee of the Zhongshan hospital, Fudan University (Approved number: B2020-275R) and performed in accordance with the Declaration of Helsinki. Written informed consent was obtained from each patient prior to the study.

### Cell lines and co-culture system

The co-culture system was established by HCC cell lines and human hepatic stellate cell line LX-2. Human hepatocellular carcinoma cell lines PLC, HCCLM3 and SMMC-7721 were obtained from the Liver Cancer Institute, Zhongshan Hospital. The HSC cell line LX-2 was a kind gift from Prof. Lieming Xu, Department of Gastroenterology, Shuguang Hospital, Shanghai University of Traditional Chinese Medicine. All cell lines were maintained in Dulbecco's modified Eagle's medium (Gibco) supplemented with 10% fetal bovine serum (Gibco) and 1% penicillin-streptomycin (Gibco) at 37℃ in a humidified incubator with 5% CO_2_.

For co-culture, LX-2 cells were seeded into the lower compartment of the Transwell chamber (0.4μm pore size, 24mm diameter, Corning Costar, MA, USA) and liver cancer cell lines were seeded into the upper chamber at a density of 10^5^ per well of a 6-well plate, respectively. Co-culture cells were maintained in DMEM with 10% FBS for 7 days with 2 or 3 times of passages and LX-2 cells were separately cultured as a negative control group.

### miRNA microarray

miRNA microarray assays were performed with an 8×12 K Agilent Human miRNA microarray (Agilent Technologies, Santa Clara, CA, USA). Total RNA was extracted using the RNeasy Mini plus Kit (Qiagen, Courtaboeuf, France) and RNA quality was evaluated by the Agilent 2100 bioanalyzer (Agilent Technologies). Array experiments were carried out according to the provider's protocols. The microarray was scanned with the Agilent array scanner and expression data were normalized by the quantile method using package limma of R software (http://www.r-project.org). The miRNAs with |log2FC|≥1 and false discovery rate (FDR) ≤0.05 were defined as differential expression and selected for further analysis.

### Exosomes isolation

The ExoQuick-TC kit (System Bioscience, SBI, USA) was used for exosome isolation from the complete medium (CM) according to the manufacturer's protocol. Detailed methods were provided in the supplementary materials of this manuscript.

### RNA interference and exosome uptake assay

Cy5-labelled hsa-mir-148a-3p mimic and mimic control were purchased from RiboBio (Guangzhou, China). Briefly, Exosomes extracted by the ExoQuick-TC were resuspended in 300μL PBS and transfected with miRNA mimics using Exo-Fect siRNA/miRNA Transfection Kit (System Biosciences, Palo Alto, CA) according to the manufacturer's protocol.

For exosome-uptaking assay, transfected exosomes were labelled with PKH67 (Sigma-Aldric, St. Louis, MO, USA) away from light at room temperature for 4min. After incubation with pre-treated exosomes for 24h, tumor cells were stained with phalloidin-rhodamine (Red, Sigma-Aldric) and DAPI (Blue, Beyotime). Images of tumor cells were captured using a laser confocal microscope (Leica, Germany) and qRT-PCR was performed to validate the miRNA expression level.

### Animal model

To investigate the roles of exosomes derived from HSCs, HCCLM3 cells transfected with luciferase lentivirus (1×10^7^) were inoculated subcutaneously into the right armpit of 4-week-old female Balb/c nude mice (Charles River laboratories, Beijing, China) housed in laminar-flow cabinets under specific pathogen-free conditions. After euthanasia of animals, tumors were procured and cut into small pieces of approximately 2 mm^3^. Subsequently, *in situ* hepatoma murine models were generated by implanting tumor pieces into the left lobe of liver. Exosomes (10 ug) extracted from the cell culture media of LX-2 were transfected with 1nmol Cy5- labelled hsa-mir-148a-3p agomir (Ribobio, Guangzhou, China) or negative control using Exo-Fect siRNA/miRNA Transfection Kit (System Biosciences, Palo Alto, CA) prior to injecting into mice through tail vein. The exosomes treatment started on day 7 and was performed every three days for four weeks. Then, all mice were euthanized and tumor tissues were cryo-sectioned and processed for fluorescent detection. All related experimental protocols were approved by the Institutional Animal Care and Use Committee at Zhongshan Hospital, Fudan University (Shanghai, China).

### Detailed materials and methods

A detailed materials and methods description were provided in the supplementary materials of this manuscript.

### Statistical analysis

Experiments were repeated at least three times and results were represented as mean±standard error unless specific indication. Comparison between two groups was performed with unpaired Student's t-test. ANOVA or Bonferroni's tests were used for multiple comparisons when appropriate. Survival analysis was performed with Kaplan-Meier analysis and examined by the log-rank test. Statistical analysis was conducted using GraphPad Prism 8.0 software (GraphPad Software, San Diego, CA, USA) and R software (v3.6.3, http://www.r-project.org). A *P* value< 0.05 was regarded as statistically significant.

## Results

### The miRNA expression profiling of HSCs co-cultured with liver cancer cells

**Figure 1:**
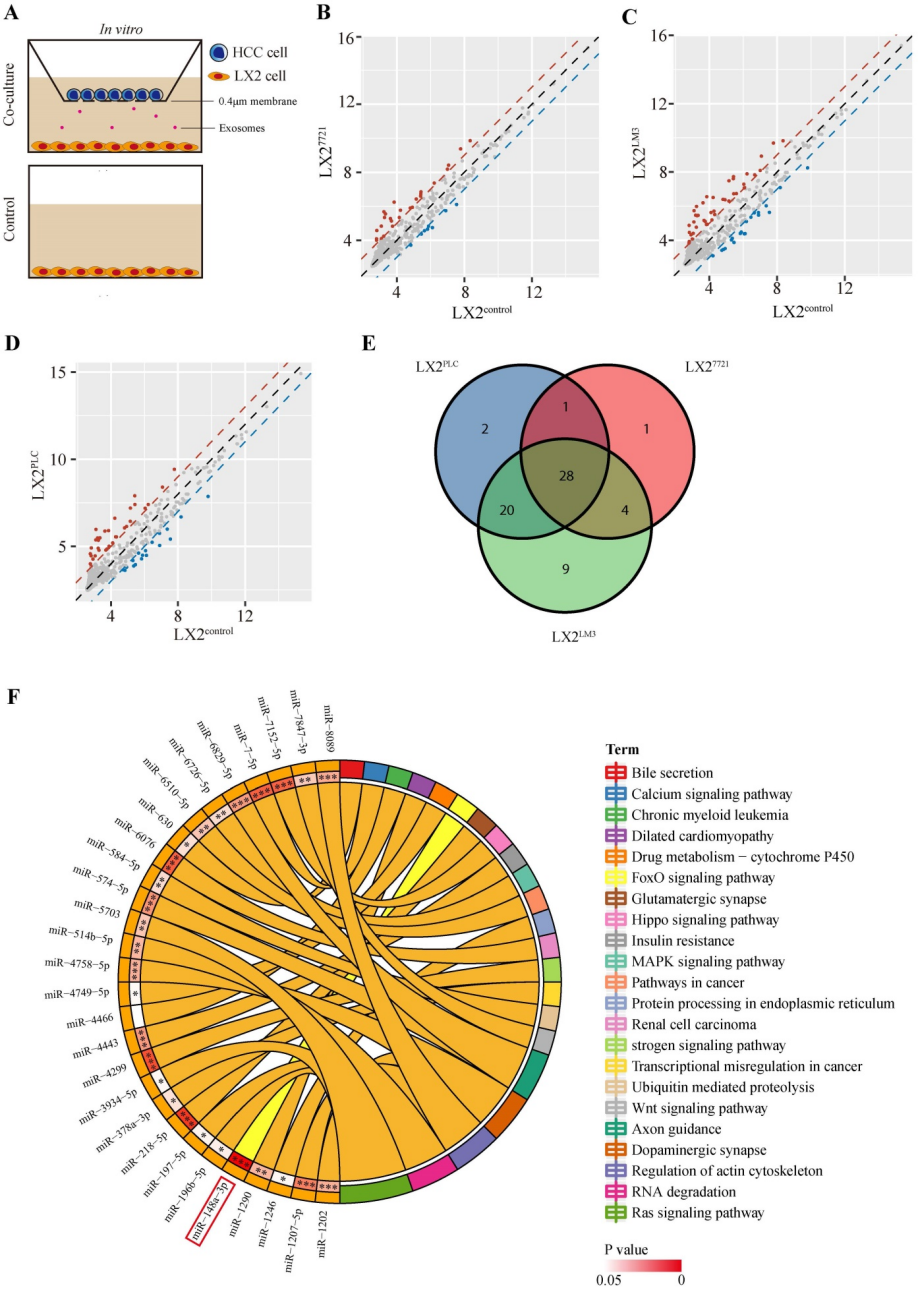
**Profiling of miRNA expression in LX-2 cells co-cultured with liver cancer cells.** (A) Illustration of the indirect co-culture system. LX-2 cells were seeded into the lower chamber and HCC cell lines were introduced into the upper chamber and co-cultured for two weeks in the 6-well plate. (B), (C) and (D) Scatter plots of differentially expressed miRNAs in LX-2 cells co-cultured with SMMC-7721, HCCLM3 and PLC, respectively. Red, upregulation; Blue, down-regulation. (E) The Venn diagram of all differently expressed miRNAs between the three groups of co-cultured LX-2 cells. (F) The KEGG pathways with the smallest *P* value of candidate miRNA target genes.

To investigate the interaction of HSCs and tumor cells in the microenvironment, we performed co-culture of human HSC cell line LX-2 with three liver cancer cell lines, PLC/PRF/5 (PLC), SMMC-7721 (7721) or HCCLM3 (LM3), respectively, for two weeks (**Figure 1A**). Then, the miRNA array analysis was performed to identify the expression profiles of co-cultured LX-2 cells. Of the 2549 miRNAs probes present in the array, 51, 34 or 61 dysregulated miRNAs were detected in LX-2 cells co-cultured with PLC, 7721 or LM3, respectively (**Figure 1B-D, Table S2**). Taking the intersection of three groups, 28 dysregulated miRNAs were obtained (**Figure 1E, Table S2**). We then performed KEGG enrichment analysis of candidate miRNA target genes and found that miR-148a-3p was significantly involved in cancer-associated pathways with the least significant difference at *P* = 2.6E-06 (**Figure 1F**) and subsequently chosen for further experiments.

### Down-regulated expression of miR-148a-3p in HSCs and primary liver cancer-associated fibroblasts

**Figure 2:**
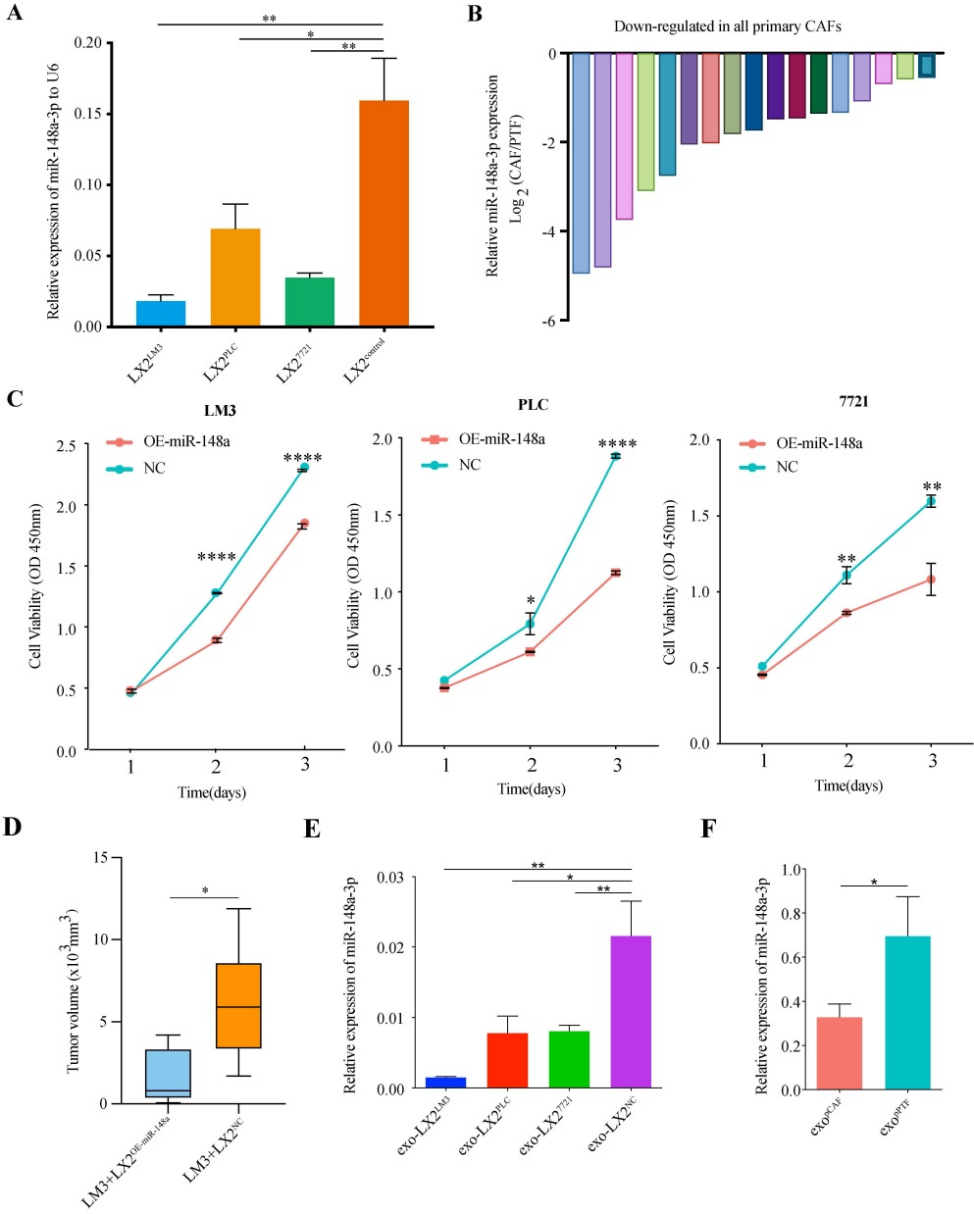
** MiR-148a-3p was down-regulated in liver cancer associated fibroblasts.** (A) The expression levels of miR-148a-3p in co-cultured LX-2 cells were significantly down-regulated compared with that of control. Data represents mean±s.d. (n=3). *P<0.05, **P<0.01. (B) Primary CAFs exhibited lower expression level of miR-148a-3p than primary PTFs in all 17 patients. (C) Cell viability was assessed by Cell Counting Kit-8 (CCK8) assay in HCC cell lines (LM3, PLC and 7721). Overexpression of miR-148a-3p significantly impaired the proliferation ability of HCC cells. Data represent mean±s.d. (n=3). *P<0.05, **P<0.01, **** P<0.0001. (D) Tumor volumes in athymic nude mice bearing subcutaneously xenografts generated from co-injected LM3 and LX-2 cells overexpressed miR-148a (LX-2^OE-miR-148a^) or control (LX-2^NC^), respectively. The miR-148a significantly inhibited tumor growth as measured by tumor volume. *P<0.05 (E) The relative expression of miR-148a-3p in exosomes derived from co-cultured LX-2 cells were significantly down-regulated compared with that of control. The miRNA expression level was normalized to U6. exo, exosome; *P<0.05; **P<0.01.(F) The expression level of miR-148a-3p in exosomes isolated from primary CAF (pCAF) was significantly down-regulated compared with that of primary PTF (pPTF). exo, exosome; *P<0.05.

Verification of miR-148a-3p expression level using real-time qRT-PCR was performed on LX-2 cells (co-cultured with PLC, 7721 or LM3, respectively). We found that the expression of miR-148a-3p was significantly reduced in co-cultured LX-2 cells compared with control (**Figure 2A**, P<0.05 for all). The HCC tumor tissues were also collected to identify the expression level of miR-148a-3p in CAFs sorted by magnetic beads after tumor tissues homogenized to single-cell suspension, as well as in fibroblasts derived from paired peritumor tissues. The primary CAFs and peritumor fibroblasts (PTFs) isolated from liver tissues were uniformly fusiform (**Figure S1A**) and determined by immunofluorescence for cell markers (**Figure S1B**). Western blotting and IHC found that the expression level of α-SMA was higher in CAFs than PTFs (**Figure S1C-D**). Of the 17 paired tissue samples detected, the expression levels of miR-148a were all significantly down-regulated in CAFs compared with that in normal fibroblasts (**Figure 2B, Figure S1E**).

### miR-148a-3p overexpression in HSCs inhibits the proliferation of co-cultured HCC cells *in vitro* and *in vivo*

To further evaluate the biological function of miR-148a-3p in HSCs on co-cultured HCC cells, LX-2 cells were transfected with miR-148a or a miRNA mimic control (**Figure S1F**) and then cocultured with HCC cell lines. The data of CCK8 assay showed that the cell viability of HCC cells was significantly suppressed after co-cultured with LX-2^OE-miR-148a^ cells compared with LX-2^NC^ (**Figure 2C**). The *in vivo* effect of HSCs on HCC was investigated using tumor models by subcutaneously co-injecting LM3 and LX-2^OE-miR-148a^ or control (LX-2^NC^) into Balb/c athymic male mice. After 4 weeks, mice co-injected LX-2^OE-miR-148a^ showed significant reduced tumor size when compared with that of LX-2^NC^ group (**Figure 2D**).

### Increased HSCs-derived exosomal miR-148a-3p suppresses HCC tumorigenic function *in vitro* and *in vivo*

We next explored how HSCs affected the function of HCC cells. Previous studies have revealed that miRNAs could be transported by the exosomes. Therefore, we hypothesized that the shift from HSCs to activated HSCs (aHSCs) led to the down-regulation of miR-148a-3p, as well as that in exosomes secreted by aHSCs, with ensuing uptake a decreased level of exosomal miR-148a-3p to induce a miR-148a-3p depletion in HCC cells, promoting cancer progression. To validate these hypotheses, HSCs-derived exosomes were isolated according to the manufacture's protocol (**Figure S2A-D**). Then, HSCs-derived exosomes were incubated with HCC cells, followed by evaluating the proliferation and invasion ability. As shown in **Figure 2E**, the low expression level of miR-148a-3p in exosomes was positively correlated with that of co-cultured LX-2 cells, as well as in exosomes secreted by patient-derived primary CAFs (**Figure 2F**).

**Figure 3:**
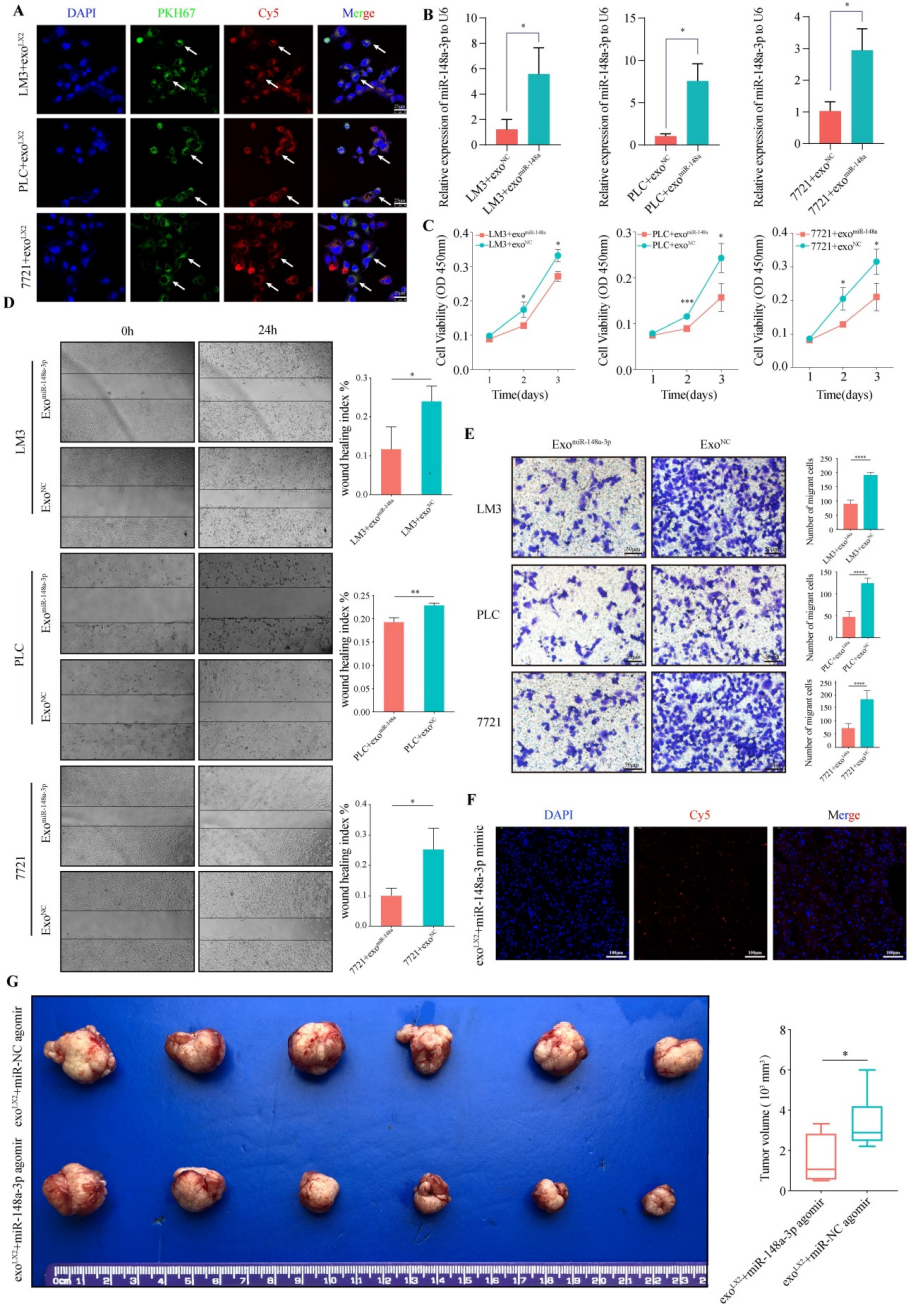
** Upregulated HSCs-derived exosomal miR-148a-3p suppresses HCC migration and invasion *in vitro* and* in vivo*.** (A) Representative images of *in vitro* uptake of PKH67-labelled exosomes (Green) loaded with Cy5-labelled miR-148a mimic (Red) by HCC cells (LM3, PLC or 7721, respectively; DAPI, blue). Scale bar, 25μm. (B) The expression of miR-148a-3p in HCC cells incubated with miR-148a-3p-loaded exosomes was significantly overexpressed compared with that of control (n=3). exo, exosome; *P<0.05. (C) CCK-8 assay revealed that liver cancer cells after incubation with miR-148a mimic loaded exosomes prominently impaired the ability of proliferation (n=3). *P<0.05, ***P<0.001. (D) and (E) Increased expression level of miR-148a-3p in exosomes inhibits migration and invasion ability of co-cultured liver cancer cells. The histogram depicts mean and standard deviation for three independent experiments. *P<0.05, **P<0.01, ****P<0.0001. Scale bar, 25μm. (F) Representative images of *in vivo* uptake of exosomes loaded with Cy5 labeled hsa-miR-148a-3p agomir (Red) by immunofluorescence analysis on tumor frozen tissue sections. Scale bar, 100μm. (G) Representative images of orthotopic tumor in nude mouse model. Volume of tumor was measured and data were presented as mean ±s.d. (n=6). Overexpressed exosomal miR-148a-3p significantly suppressed orthotopic HCC proliferation. *P<0.05.

Uptake of exosomes by HCC cells were observed after 24h incubation with LX-2 exosomes loaded with miR-148a-3p mimic conjunct with cy5 (Red) and pre-stained with PKH67 (Green). Using confocal microscopy, exosomes were captured coexisted with miR-148a-3p and located in cytoplasm of HCC cells (**Figure 3A**). We also found that HCC cells co-cultured with miR-148a-3p-loaded exosomes exhibited an increased level of miR-148a-3p compared with controls (**Figure 3B**). To investigate whether exosomal miR-148a-3p can exert tumor suppressor effect after taken up by HCC cells, the proliferation and invasive ability were also determined. As shown in **Figure 3C-E**, miR-148a-3p mimic loaded exosomes significantly inhibited the proliferation ability of tumor cells, as well as the cell motility and invasion.

To further confirm the anti-tumor effect of exosomal miR-148a derived from HSCs *in vivo*, exosomes isolated from the medium of LX-2 cells were transfected with Cy5 labeled hsa-miR-148a-3p agomir or agomir NC and subsequently injected into the orthotopic liver tumor bearing BALB/c mice for 4 weeks via tail vein, with each mouse receiving 100 μL of exosomes three times a week. Assessment of tumor frozen section using the scanning laser confocal microscope showed that the Cy5 signal of agomir could be detected, indicating exosomes were uptaked by tumor cells (**Figure 3F**). As expected, overexpressed exosomal miR-148a-3p significantly suppressed hepatoma cells proliferation (**Figure 3G**).

### miR-148a-3p acts as tumor suppressor and inhibits HCC cells malignancy by targeting ITGA5 through PI3K/Akt signaling

**Figure 4:**
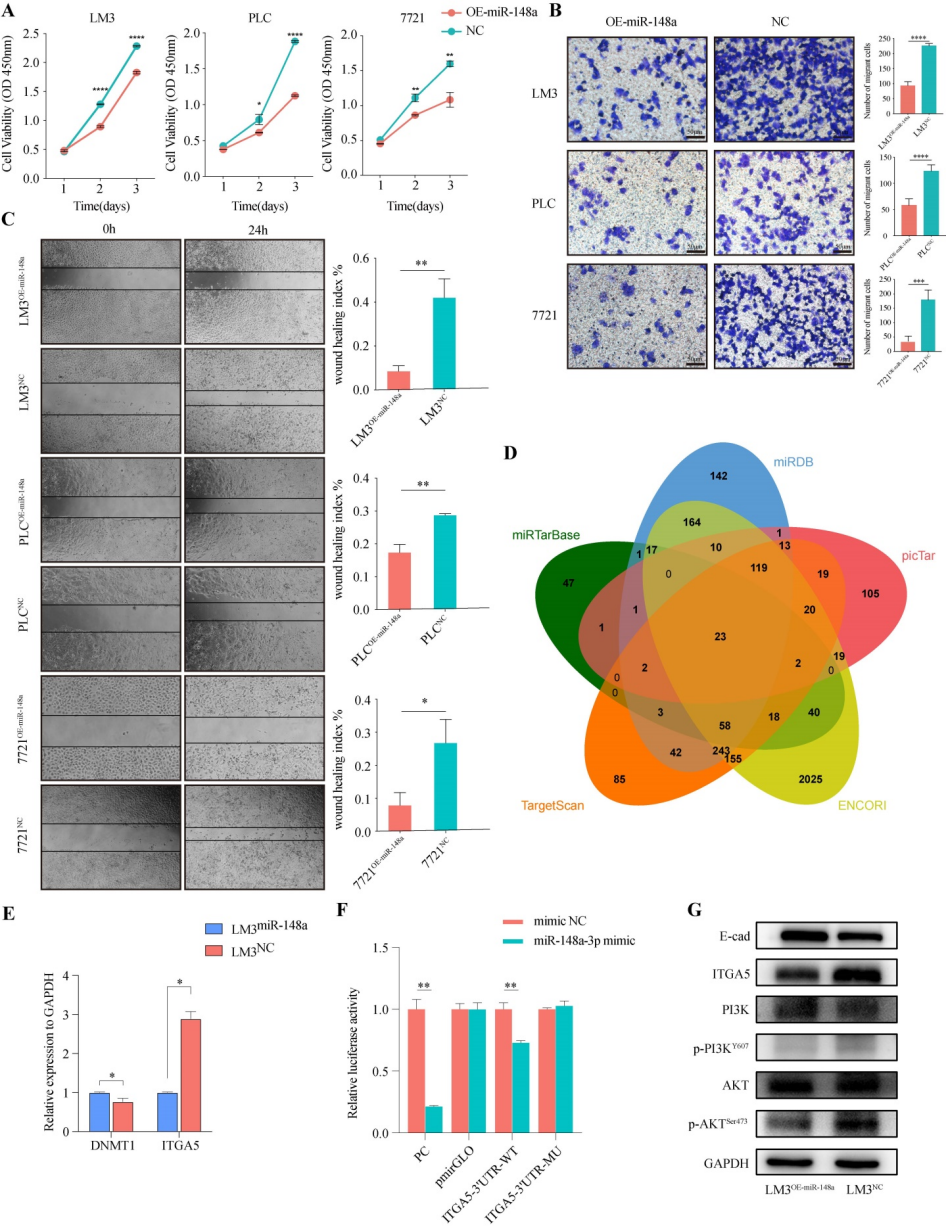
** MiR-148a-3p acts as tumor suppressor and inhibits HCC cells malignancy by targeting ITGA5 through PI3K/Akt pathway.** (A) Cell viability was evaluated in miR-148a or control transfected human HCC cells (LM3, PLC or 7721, respectively) using CCK-8 assay. Data represent the mean with s.d. (n=3). *P<0.05, **P<0.01, ****P<0.0001. (B) Invasion assays for indicated HCC cells were performed by transwell inserts with Matrigel. Scale Bar, 50μm. ***P<0.001, ****P<0.0001. (C) Representative images of wound healing assay of HCC cells transfected with miR-148a-3p as monitored 24 h after scratching. Wound-healing index was quantified. Data represent mean with s.d. (n=3). *P<0.05, **P<0.01. (D) Venn diagram showing the overlap of predicted target genes of miR-148a-3p in 5 miRNA target prediction databases (miRDB, picTar, ENCORI, TargetScan, miRTarBase). (E) The expression level of ITGA5 was significantly down-regulated in LM3 cells over-expressed miR-148a-3p. (F) Dual luciferase reporter assay in HEK293T cells co-transfected with firefly luciferase constructs containing the wild type or mutant miR-138a-3p site of ITGA5 3'UTR and firefly luciferase activity was normalized to Renilla luciferase (n=3). WT, wild type; Mu, mutant; **P<0.01. (G) miR-148a-3p impaired malignancy of HCC cells through inhibiting ITGA5/PI3K/Akt signaling.

To evaluate the function of miR-148a-3p in HCC cells, miR-148a overexpressing lentivirus or control was transfected into PLC, 7721 and LM3, respectively. We assess the effect of miR-148a on cell growth using CCK-8 assay. As shown in **Figure 4A**, overexpression of miR-148a significantly suppressed cell proliferation in HCC cells. Also, the results of Transwell and wound-healing assay suggested that overexpression of miR-148a inhibited the invasive and migrative ability of tumor cells (**Figure 4B-C**).

To further investigate the potential mechanisms by which miR-148a-3p suppresses HCC progression, we searched for candidate target genes of miR-148a-3p in 5 miRNA target prediction databases (miRDB, miRTargetBase, miRanda, TargetScan, picTar) (**Figure 4D**). Of listed 23 predicted target genes, ITGA5 and DNMT1 were significantly dysregulated based on the TCGA-LIHC dataset and selected for further studies (**Figure S3**). Next, we performed qRT-PCR analysis to validate the expression of target gene and found that ITGA5 was consistent with the expression change of miR-148a-3p (**Figure 4E**). Moreover, a dual-luciferase reporter gene assay was performed in 293T cells and the results demonstrated that overexpression of miR-148a-3p significantly reduced the firefly luciferase activity of the ITGA5-3'UTR-WT compared with that of ITGA5-3'UTR-MU (**Figure 4F, Figure S4**). The results obtained thus far indicated that PI3K/Akt signal was required for tumorigenesis of HCC cells induced by the miR-148a-3p/ITGA5 axis. Therefore, we evaluated levels of PI3K and Akt and their phosphorylated status in HCC cells followed by stable upregulation of miR-148a-3p expression. Our results showed significant differences in the level of ITGA5, E-cad, phosphorylated PI3K (P-PI3K) and phosphorylated Akt (P-Akt) (**Figure 4G**). These findings indicated that miR-148a-3p/ITGA5 axis induced the activation of PI3K/Akt signal and promoted the malignancy of HCC cells through EMT.

### miR-148a-3p and ITGA5 correlates with prognosis of HCC patients

**Figure 5:**
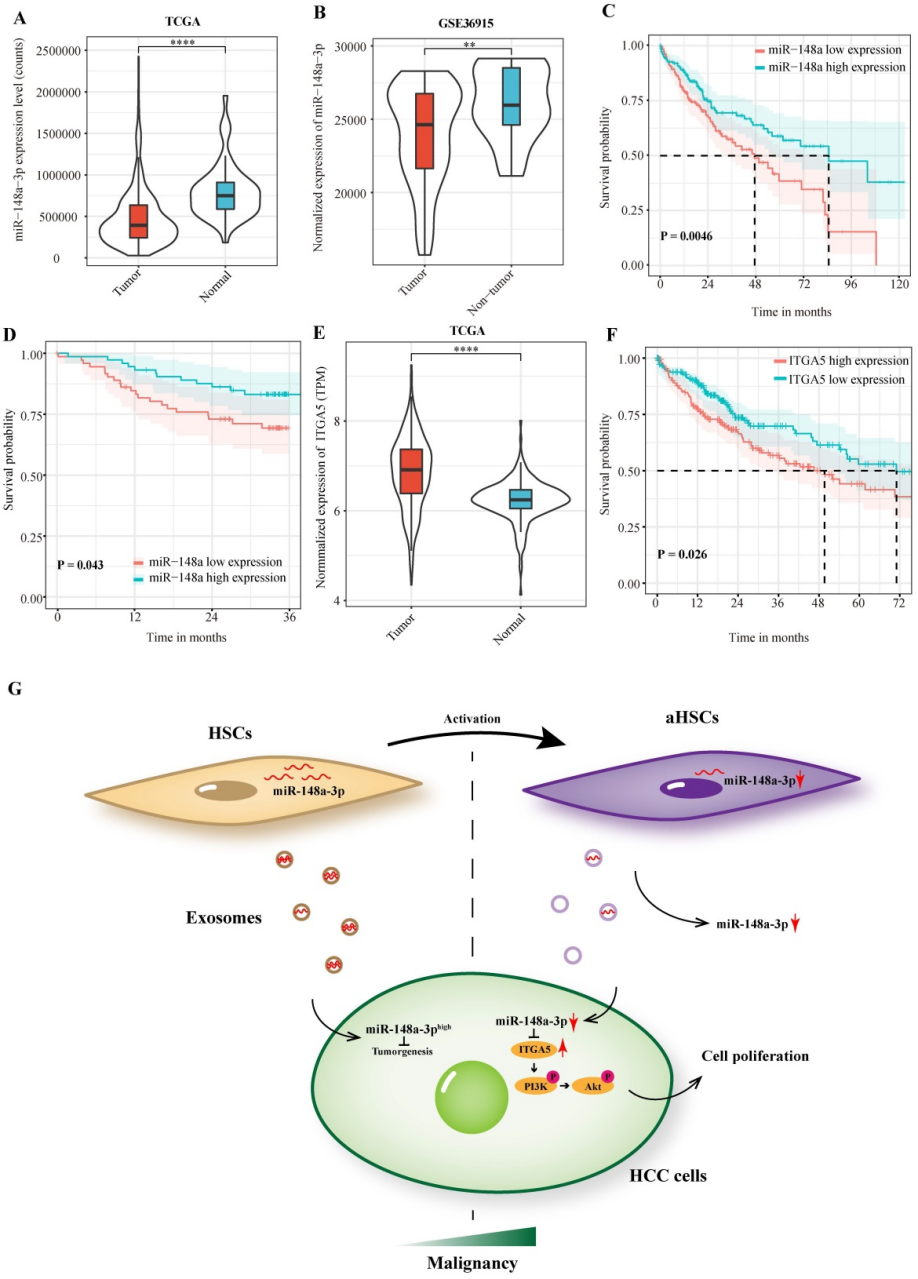
** MiR-148a-3p and ITGA5 correlates with prognosis of HCC patients.** (A)Analysis of miR-148a-3p expression in the HCC tumor samples using TCGA (The Cancer Genome Atlas) database. ****P<0.0001. (B) MiR-148a-3p was significantly down-regulated in tumor tissues in the GEO database (GSE36915). ** P<0.01. (C) Kaplan Meier survival analysis of miR-148a-3p was performed for overall survival in 373 patients with HCC. (D) Overall survival analysis based on the expression level of miR-148a-3p in plasma samples. (E) Analysis of ITGA5 expression in the HCC tumor samples using TCGA database. ****P<0.0001. (F) Kaplan Meier survival analysis of ITGA5 in the TCGA database. (G) Schematic representation of the major molecular mechanism of exosome-depleted miR-148a-3p derived from activated HSCs contributes to tumor progression through ITGA5/PI3K/Akt in HCC. HSCs, hepatic stellate cells; aHSCs, activated hepatic stellate cells.

In order to investigate the clinical significance of miR-148a-3p expression in patients with HCC, we performed a preliminary analysis using the TCGA-LIHC dataset. The results showed that miR-148a-3p was down-regulated in liver cancer tissues compared with normal tissues, which was validated by the GSE 36915 dataset (**Figure 5A-B**). Patients were divided into high- and low- expression subgroups according to the median expression of miR-148a-3p in tumor tissues. The result demonstrated that patients with low miR-148a-3p expression has a significantly shorter OS time than compared with high expression group (P = 0.005, **Figure 5C**). Survival analysis was subsequently performed on the plasma samples of 146 HCC patients in Zhongshan hospital. Consisted with TCGA data, patients with low miR-148a-3p expression in plasma has a significantly shorter OS time (P = 0.043, **Figure 5D**). We also investigated the expression of ITGA5 in patients with HCC using TCGA database and found that ITGA5 was significantly upregulated in tumor tissues, which associated with poor OS in HCC patients (**Figure 5E-F**).

## Discussion

As a dynamic network system orchestrated by intercellular communication,^23^ the tumor microenvironment is emerging as a crucial determinant for tumor progression, metastasis and drug resistance.^24^ Cancer-associated fibroblasts (CAFs), the dominating source of collagen-producing cells, are the major type of stromal cells in the tumor microenvironment with distinct tumorigenic properties.^25^ Increasing evidence has indicated that crosstalk between tumor and CAFs in the TME is pivotal in tumorigenesis. Therefore, it is essential to investigate the underlying mechanisms in cell-to-cell communications mediated by exosomes. In the present study, we used a TME-like co-culture system of liver cancer cells and HSCs *in vitro*, followed by miRNA expression profiles analysis between aHSCs and control. Then, we identified that miR-148a-3p was significantly down-regulated in aHSCs as well as the HSCs-derived exosomes and promoted HCC development by through ITGA5/PI3K/Akt pathway.

Previous studies have shown that miR-148a-3p is down-expressed in CAFs and promotes tumor progression in different cancers. In a study on endometrial cancer, miR-148a expression was decreased in CAFs, which contributed to tumor cell motility.^26^ It is also determined that over-expression of miR-148a in CAFs significantly impaired the invasive and migrative ability of oral carcinoma.^27^ Hsin-Jung Wu *et al* have shown that miR-148a derived from CAFs decreased mammary tumor functions and metastasis through FAK signaling.^28^ Our data have indicated that low miR-148a-3p expression in CAFs has a positive correlation with that in exosomes, which promoted the proliferation and invasion of co-cultured HCC cells. Moreover, several studies have primarily focused on the role of miR-148a-3p in HCC development. It is well documented that miR-148a acts as an inhibitor of the IκB kinase alpha/NUMB/NOTCH pathway in promoting HCC initiation and progression.^29^ Lian Li *et al* have reported that miR-148a could suppress the expression of AVCR1, a regulator of the BMP/Wnt signaling pathway, which is important for cancer stem cells.^30^ MiR-148a was also reported to play a critical role in HBx/ URG11-mediated HCC through modulation of β-catenin and PTEN/AKT pathway^31^ and may be associated with metastasis related to HCC.^32^ Our experiments showed that over-expression of miR-148a-3p significantly impaired the proliferation, migration and invasion through targeting ITGA5. Furthermore, accumulating studies have revealed correlations between miR-148a and prognosis of HCC patients. Min Jeong Heo *et al* found miR-148a dysregulated discriminated the overall survival and recurrence of survival in HCC.^33^ It was also reported that down-expression of miR-148a was correlated to poor prognosis of HCC patients.^34^ These reports are consistent with our results indicating the inhibitory role of miR-148a-3p in HCC development and negative association with survival. Taken together, these data suggested that miR-148a-3p acts as a tumor suppressor in hepatocarcinogenesis.

Exosomes are emerging as one of the key players in the crosstalk of cells in the tumor environment, with the ability to deliver complex information to recipient cells,^35^, ^36^ A previous study has demonstrated that down-regulation of exosomal miR-320a derived from CAFs contributed to the proliferation and metastasis of hepatoma.^14^ In the present study, we employed a TME-like co-culture system of liver cancer cells and aHSCs, in which the intracellular communication was indirect through the Transwell chamber. Our results showed that the miR-148a-3p was down-expressed in exosomes secreted by co-cultured LX-2 cells, which is consistent with that in LX-2 cells. Moreover, we also confirmed that the miR-148a-3p was down-regulated in co-cultured HCC cells and clinical samples. Therefore, we hypothesized that hepatic satellite cells exhibit a high miR-148a-3p expression level in normal microenvironment, as well as HSC-derived exosomes. After uptake by hepatocytes, exosomal miR-148a-3p plays a key role in suppression of tumorigenesis. Our results demonstrated that miR-148a-3p was down-regulated in the transformation process of HSCs into aHSCs, followed by the depletion of exosomal miR-148a-3p. As an exogenous tumor suppressor factor, decreased exosomal miR-148a-3p was uptaked by HCC cells, resulting in cellular miR-148a-3p depletion and HCC development.

There have been substantial researches undertaken on the therapeutic role of exosomes.^37^, ^38^ Prior study has noted that exosomal drug delivery has the potential to enhance the effectiveness of treatment and more importantly, to reduce side effects due to higher stability and specific targeting.^39^ Additionally, recent animal experiments revealed that exosomes exhibited hepatotropic characteristics,^15^, ^40^ which further illustrated its suitableness for HCC treatment. Our results indicated that miR-148a-3p overloaded exosomes uptaked by HCC cells played a critical role in suppressing tumor development, which is expected to be a promising approach for the treatment of HCC (**Figure 5G**).

In conclusion, our results indicated the innovative role of miR-148a-3p in regulating the proliferation and invasion of HCC cells by exosomes in TME, and this study also provides a new mechanism underlying cell-cell communications in TME.

## Supplementary Material

Supplementary methods, figures and tables.
