# Supplementary material for: Exosome-depleted MiR-148a-3p derived from Hepatic Stellate Cells Promotes Tumor Progression via ITGA5/PI3K/Akt Axis in Hepatocellular Carcinoma
Source: Int J Biol Sci. 2022 Mar 6;18(6):2249–60. doi: 10.7150/ijbs.66184 (PMC8990464; doi:10.7150/ijbs.66184)
Supplement: Supplementary file 1 — Supplementary methods, figures and tables. [file ijbsv18p2249s1.pdf]

Materials and methods

Primary fibroblasts isolation

Briefly, liver tissues were dissociated and homogenized using gentleMACS C tubes (Miltenyi Biotec, Bergisch Gladbach, Germany) and the gentleMACS dissociator (Miltenyi Biotec) with a human tumor dissociation kit (Miltenyi Biotec) according to the manufacturer's instruction. After filtering through the 40μm strainer (Miltenyi Biotec) and incubating with anti-fibroblast magnetic beads (Miltenyi Biotec), fibroblasts were positively selected by magnetic sorting from single-cell suspension with LS columns (Miltenyi Biotec) and a MACS separator (Miltenyi Biotec). The purified fibroblasts suspension was then centrifuged at 300g for 10 minutes at 4°C and the pellets were cultured in Dulbecco's Modified Eagle Medium/Nutrient Mixture F-12 (DMEM/F12, Gibco, Thermo Fisher Scientific, MA, USA) with 10% fetal bovine serum (Gibco) in an atmosphere of 5% CO_2 at 37°C. In this study, the first to fifth passages of primary cells were applied for all the experiments.

Western blotting

Whole-cell protein was extracted from the cells using radio-immunoprecipitation assay (RIPA) buffer with protease inhibitors and separated by 10% sodium dodecyl sulfate-polyacrylamide gel electrophoresis (SDS-PAGE). The proteins were then transferred to PVDF membranes, followed by blocking in 5% bovine serum albumin (BSA) and incubation with specific primary antibodies overnight at 4°C. After washing in TBST buffer, the immunocomplexes were incubated with HRP-conjugated secondary antibody and the antigen-antibody bands were visualized by enhanced chemiluminescence assay (Millipore Corporation, Billerica, MA, USA). The primary antibodies and dilutions used were listed in Supplementary Table 2.

Immunofluorescence

Cells were cultured directly on chamber slides for 48h. After washing with phosphate-buffered saline (PBS), cells were fixed with 4% paraformaldehyde at room temperature for 30mins and permeabilized with 0.1% Triton X-100. After washing three times in PBS for 5 min, fixed cells were immunostained with primary antibody at 4°C overnight and subsequently incubated with Alexa Fluor 488 Goat anti-rabbit IgG. The nuclei were labeled with the dye DAPI (Invitrogen) and representative images were acquired with a fluorescence microscope (Olympus, Japan)

RNA extraction and qRT-PCT

Total cellular RNA was extracted using the RNeasy Mini plus Kit (Qiagen, Courtaboeuf, France) according to the manufacturer's protocol. Briefly, cultured cells were lysed in 600μL buffer RLT and homogenized for 1min using a rotor homogenizer. After adding same volume of 70% ethanol, the lysate was subsequently transferred to a RNeasy Mini spin column and centrifuged for 1min at 12000 rpm. Then, the spin column was washed using buffer RW1 and RPE and centrifuged for 1 min at 12000 rpm to elute the total RNA with 30μL RNase-free water. The RNA was quantified by a Nanodrop 2000 spectrophotometer (Thermo Fisher Scientific, USA) and stored at -80°C.

Synthesis of cDNA was performed using the PrimeScript RT Master Mix (Takara Bio, Japan) and qRT-PCR analysis was performed with TB Green Premix Ex Taq II

(Takara Bio, Japan) on an Applied Biosystems 7500 Fast Real-Time PCR System (Applied Biosystems, USA) according to the provider's protocols. Briefly, the amplification was carried out with a thermal cycler under the following conditions: initial holding stage at 95℃ for 30 sec, 40 cycles of 95℃ for 5 sec and 60℃ for 34 sec, followed by the melt curve stage to confirm the specificity of the products. Primers were produced by Sangon Biotech (Shanghai, China) and sequences were shown in supplementary materials. Gene expression was normalized to the housekeeping gene GAPDH and comparative Ct method ($2^{-\Delta\Delta Ct}$) was used for relative quantification.

The MagMAX mirVana Total RNA Isolation Kit (Applied Biosystems) was used for RNA extraction from cellular exosomes according to the manufacturer's instructions. After quantification, total RNA was stored at -80℃ for subsequent procedures.

Poly(A) tailing, cDNA synthesis and qRT-PCR for cellular and exosomal miRNA were performed using miDETECT A Track miRNA qRT-PCR Starter Kit (RiboBio Co., Ltd., Guangzhou, China) according to the provider's instructions. The expression levels of target miRNAs were normalized to internal reference U6 and calculated with $2^{-\Delta\Delta Ct}$ method. The primers and probes were listed in the Supplemtary Table 3 and 4.

Exosomes isolation

The ExoQuick-TC kit (System Bioscience, SBI, USA) was used for exosome isolation from the complete medium (CM) according to the manufacturer's protocol. Briefly, 5×10^6 cells were seeded into T75 flask and cultured for 48h in DEME supplemented with 10% exosome-depleted FBS (Gibco, USA). Next, CM was collected and consecutively centrifugated at 400g for 10min, 2000g for 10min and 10000g for 30min to remove cells and debris. The supernatant was then concentrated through 0.22μm filters (Millipore, USA) and pipetted into a sterile EP tube, followed by mixing with equal volume of ExoQuick-TC. After incubation overnight at 4℃, the compound was centrifuged at 3000g for 60min. The supernatant was removed, and the pellet was resuspended with ice-cold PBS for further experiment.

Exosomes characterization

Nanoparticle tracking analysis (NTA) was performed in vitro using a NanoSight NS500 instrument (Malvern Instruments, Malvern, United Kingdom) for assessing the size distribution and concentration of exosomes according to the manufacturer's instructions. Briefly, isolated exosomes were resuspended in 20μL PBS and diluted 1000-fold for further experiments. After setting the detection threshold at 3, each sample was analyzed in triplicate through the camera setting 13 with acquisition time of 30s. The NTA software (version 3.1) was used to capture and analyze the results.

For transmission electron microscopy (TEM) assay, prepared exosome pellets were fixed with 2% paraformaldehyde and 2.5% glutaraldehyde in PBS overnight at 4℃. After rinsing, a droplet of samples (5μL) was placed on formvar-coated copper grids and stained with 1% uranyl acetate. The exosomes were visualized using a Philips CM 10 TEM (Philips, Eindhoven, Netherlands) at 80kv.

Construction of stable cell lines

All recombinant lentiviruses were designed and provided by Genechem (Shanghai, China). Briefly, target and negative control miRNA sequences were synthesized and subcloned into the lentiviral vector GV209 by the restriction enzymes Age I and EcoR

I. Subsequently, HEK293T cell were co-transfected with recombinant plasmid GV209, pHelper 1.0 and pHelper 2.0 using the Lipofectamine 2000 (Invitrogen, Thermo Fisher Scientific, USA). Target cells were infected with the purified lentivirus in a multiplicity of infection (MOI) of 40 for 48h and selected with puromycin for 7 days. The transfection efficiency was measure by the GFP and the expression level of miR-148a-3p was confirmed by qRT-PCR analysis.

Cell proliferation, migration and invasion assays

Cell viability was measured by the Cell Counting Kit 8 (Dojindo, Limamoto, Japan) assay according to the manufacturer's instructions. In brief, cells were seeded into a 96-well plate with 6 independent repeats at 2×10^3 per well. After culturing for 24h, 48h and 72h, CCK-8 reagent (10μL per well) was added for additional two hours incubation at 37°C with 5% CO2, respectively. Cell proliferation rate was determined by detecting the optimal absorbance at 450nm.

For cell migration assay, 1×10^6 transfected cells were planted into a 6-well plate and cultured for 24h. Adherent cell monolayers were then wounded with a sterile pipette tip to create a linear gap. The migration ability in terms of wound healing was analyzed using Image J software based on the photographs captured at the time of wounding and 24 hours later. Wound healing index was calculated as: (original wound area - actual wound area)/original wound area. The expriments were performed in triplicate.

Cell invasion assay was performed using Transwell chambers with 8-μm pore size polycarbonate membranes (Corning, NY, USA) in 24-well plates. Briefly, starvation induced cells were digested, centrifuged and resuspended in DMEM with 1% BSA. Then, transfected cells (1×10^5) were seeded into the upper chambers coated with 100μL Matrigel (BD Biosciences, San Jose, CA, USA). The lower chambers were filled with 600μL DMEM supplemented with 10% FBS. After incubation at 37°C for 48h, non-invasive cells in the upper chambers were removed and cells invaded the lower surface of the membrane were rinsed with PBS, fixed in 4% paraformaldehyde for 30min and stained with 0.1% crystal violet. Invasion ability was quantified by counting migration cells at five randomly selected fields (×200 magnification) of each well with an optical microscope (Olympus, Japan).

Luciferase reporter gene assay

The HEK293T cells were seeded in 24-well plates at a density of 2×10^4 per well and cultured to 60% confluency. Subsequently, the cells were co-transfected with Wide/Mutant type pmirGLO-h-ITGA5-3'UTR plasmids (1μg) and miR-148a-3p mimics/negative control (60nm) using X-tremegene HP (Roche). After transfection for 48h at 37°C, luciferase activity was detected with a Dual-Luciferase Reporter Assay System (Promega Corporation, Madison, WI, USA) according to the manufacturer's protocol. Relative luciferase activity was standardized to the renilla luciferase activity.

Table S1. Inclusion and Exclusion Criteria

	**Inclusion and Exclusion Criteria**
A	The diagnosis of HCC was confirmed pathologically, intrahepatic cholangiocellular carcinoma and mixed liver cancer were excluded.
B	None of the patients had received previous treatment for HCC.
C	No history of other malignancies

Table S2. MicroRNA expression profiles of co-cultured LX2 cells

	LX2^{PLC}	LX2^{7721}	LX2^{LM3}
**Upregulated**	hsa-miR-1202*	hsa-miR-1202	hsa-miR-1202
	hsa-miR-1207-5p*	hsa-miR-1207-5p	hsa-miR-1207-5p
	hsa-miR-1246*	hsa-miR-1246	hsa-miR-1246
	hsa-miR-1290*	hsa-miR-1290	hsa-miR-1268a
	hsa-miR-197-5p*	hsa-miR-197-5p	hsa-miR-1290
	hsa-miR-3173-3p	hsa-miR-3934-5p	hsa-miR-197-5p
	hsa-miR-3679-5p	hsa-miR-4299	hsa-miR-3173-3p
	hsa-miR-3934-5p*	hsa-miR-4466	hsa-miR-3679-5p
	hsa-miR-4259	hsa-miR-4530	hsa-miR-3934-5p
	hsa-miR-4271	hsa-miR-4749-5p	hsa-miR-4271
	hsa-miR-4298	hsa-miR-514b-5p	hsa-miR-4281
	hsa-miR-4299*	hsa-miR-5703	hsa-miR-4298
	hsa-miR-4430	hsa-miR-574-5p	hsa-miR-4299
	hsa-miR-4442	hsa-miR-5787	hsa-miR-4430
	hsa-miR-4466*	hsa-miR-584-5p	hsa-miR-4442
	hsa-miR-4740-5p	hsa-miR-6076	hsa-miR-4466
	hsa-miR-4749-5p*	hsa-miR-6087	hsa-miR-4530
	hsa-miR-483-5p	hsa-miR-630	hsa-miR-4740-5p
	hsa-miR-514b-5p*	hsa-miR-642b-3p	hsa-miR-4749-5p
	hsa-miR-5703*	hsa-miR-6510-5p	hsa-miR-483-5p
	hsa-miR-574-5p*	hsa-miR-6726-5p	hsa-miR-514b-5p
	hsa-miR-584-5p*	hsa-miR-6829-5p	hsa-miR-5703
	hsa-miR-6076*	hsa-miR-7152-5p	hsa-miR-574-5p
	hsa-miR-6165	hsa-miR-7847-3p	hsa-miR-5787
	hsa-miR-630*	hsa-miR-8089	hsa-miR-584-5p
	hsa-miR-6510-5p*	hsa-miR-877-5p	hsa-miR-6076
	hsa-miR-6726-5p*		hsa-miR-6087
	hsa-miR-6738-5p		hsa-miR-6165
	hsa-miR-6749-5p		hsa-miR-630
	hsa-miR-6829-5p*		hsa-miR-642b-3p
	hsa-miR-7152-5p*		hsa-miR-6510-5p
	hsa-miR-7847-3p*		hsa-miR-6726-5p
	hsa-miR-8089*		hsa-miR-6749-5p
			hsa-miR-6763-5p
			hsa-miR-6793-5p
			hsa-miR-6829-5p
			hsa-miR-6879-5p
			hsa-miR-7152-5p
			hsa-miR-7845-5p
			hsa-miR-7847-3p
			hsa-miR-8089

**Downregulated**	hsa-miR-1260a	hsa-miR-148a-3p	hsa-miR-10b-5p
	hsa-miR-1260b	hsa-miR-196b-5p	hsa-miR-1260a
	hsa-miR-140-3p	hsa-miR-19a-3p	hsa-miR-1260b
	hsa-miR-146b-5p	hsa-miR-218-5p	hsa-miR-140-3p
	hsa-miR-148a-3p*	hsa-miR-378a-3p	hsa-miR-146b-5p
	hsa-miR-185-5p	hsa-miR-4443	hsa-miR-148a-3p
	hsa-miR-196a-5p	hsa-miR-4758-5p	hsa-miR-185-5p
	hsa-miR-196b-5p*	hsa-miR-7-5p	hsa-miR-196a-5p
	hsa-miR-199a-3p		hsa-miR-196b-5p
	hsa-miR-19a-3p		hsa-miR-199a-3p
	hsa-miR-218-5p*		hsa-miR-218-5p
	hsa-miR-28-5p		hsa-miR-28-5p
	hsa-miR-378a-3p*		hsa-miR-340-5p
	hsa-miR-378i		hsa-miR-374b-5p
	hsa-miR-4443*		hsa-miR-378a-3p
	hsa-miR-4758-5p*		hsa-miR-378i
	hsa-miR-7-5p*		hsa-miR-4443
	hsa-miR-92a-3p		hsa-miR-4758-5p
	hsa-miR-1260a		hsa-miR-7-5p
			hsa-miR-92a-3p

*means repeated miRNAs in all three groups.

Table S3. Demographic and clinical characteristics of patients

**Clinical characteristics**	**Number of patients**
Age (years)	
≤60	7
> 60	9
Sex	
Male	12
Female	4
Tumor number	
Single	12
Multiple	4
Tumor size (cm)	
≤5	9
> 5	7
Hepatitis B	
Positive	15
Negative	1
TNM stage (8th AJCC)	
I	8
II	2
III	5
IV	1
Overall	16

Table S4. The primary antibodies and dilutions

Antibody	Manufacturer	Catalog	Dilution
Anti-Integrin alpha 5 mAb	Abcam	ab150361	1:5000
E-Cadherin (24E10) Rabbit mAb	CST	3195S	1:1000
Anti-PI 3 Kinase p85 alpha mAb	Abcam	ab191606	1:1000
Anti-PI 3 Kinase p85 alpha (phospho Y607) pAb	Abcam	ab182651	1:1000
Anti-pan-AKT mAb	Abcam	Ab8805	1:500
Phospho-Akt (Ser473) (D9E) XP® Rabbit mAb	CST	4060S	1:1000
Anti-GAPDH mAb	Abcam	ab8245	1:5000

Table S5. Primer sequences

Gene	Forward sequences (5' to 3')	Reverse sequences (5' to 3')
GAPDH	GGGGCTCTCCAGAACATCATCC	ACGCCTGCTTCACCACCTTCTT
ITGA5	GGTCGGGGGCTTCAACTTA	GAGCCGAGAGCCTTTGCTG

Table S6. Probe sequences

Assay ID	Assay Name	Target Sequence
001973	U6 snRNA	5'-GUGCUCGCUUCGGCAGCACAUAUACUAAAA UUGGAACGAUACAGAGAAGAUUAGCAUGGCC CCUGCGCAAGGAUGACACGCAAAUUCGUGAA GCGUUCCAUAUUUU-3'
000470	hsa-miR-148a-3p	5'-UCAGUGCACUACAGAACUUUGU-3'

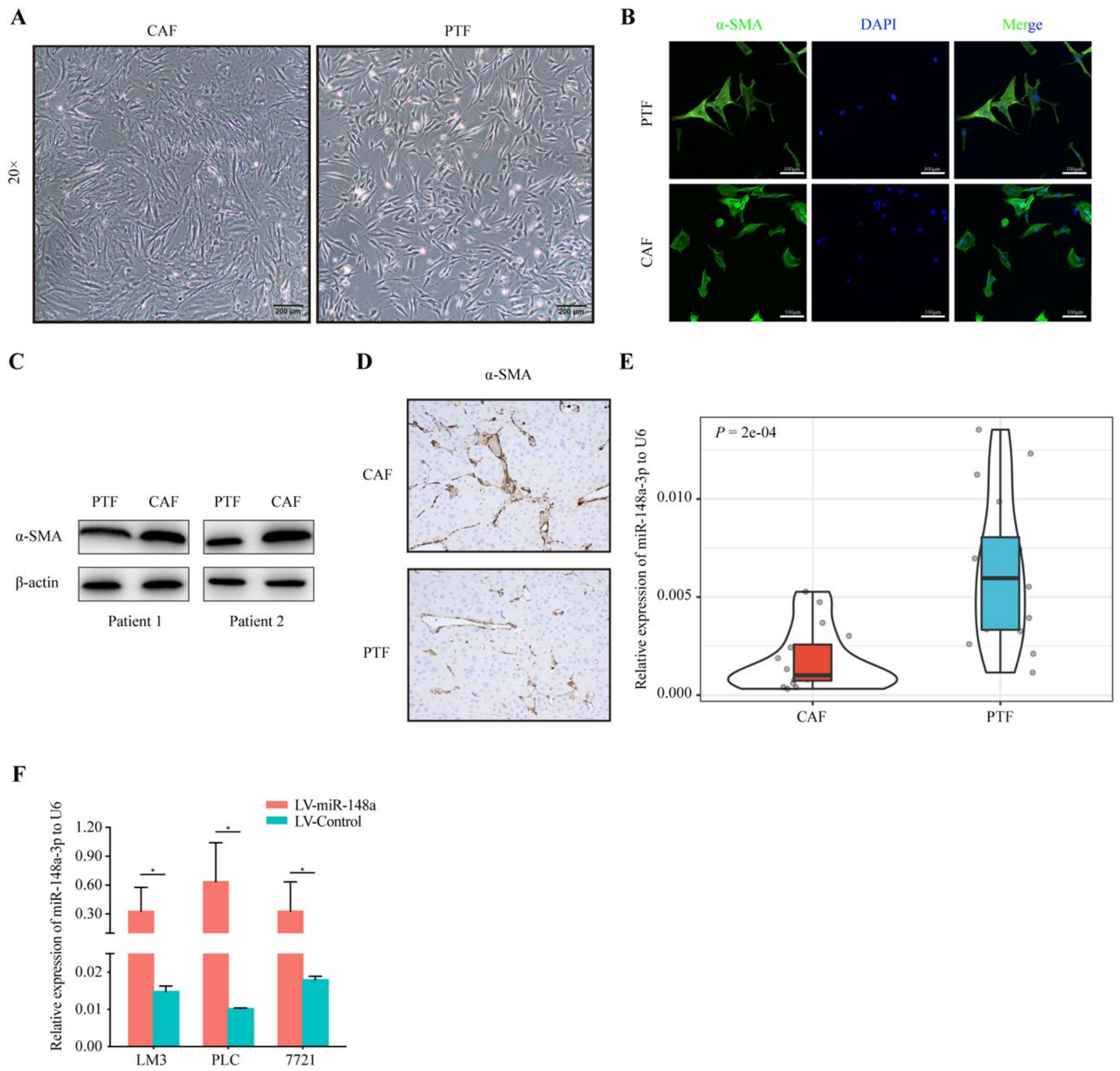

Figure S1. Isolation and characterization of primary fibroblasts.

(A) Representative images of primary cancer-associated fibroblast (CAF) and peritumor fibroblast (PTF) (magnification 20×). (B) Representative images show expression of the activated fibroblast marker α-SMA in CAF and PTF (α-SMA, Green; DAPI, Blue). (C) Primary CAFs exhibited higher expression of α-SMA than that of PTFs. (D) Representative IHC staining images of α-SMA expression in CAF and PTF. (E) Primary CAFs exhibited lower expression level of miR-148a-3p than primary PTFs in 16 patients. (F) MiR-148a-3p expression quantified by qPCR in HCC cells transfected with either miR-148a or empty vector. (*$P<0.05$. LV, lentivirus)

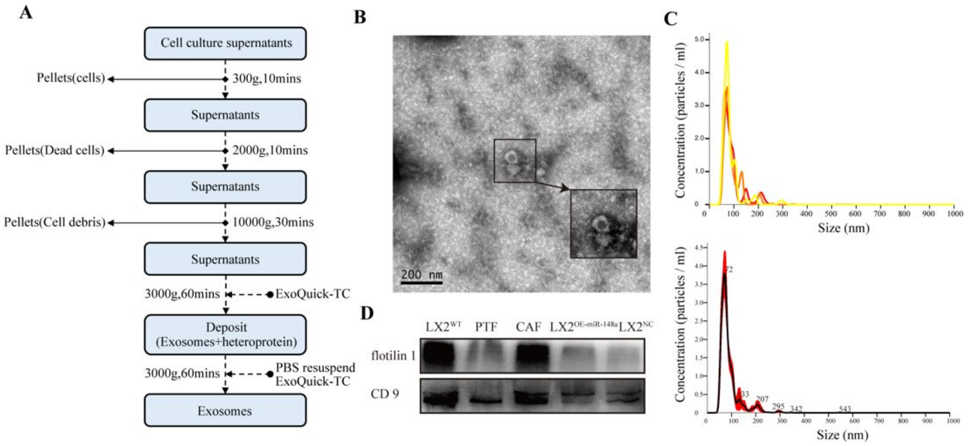

Figure S2. Isolation and characterization of exosomes derived from cancer associated fibroblasts.

(A) Flow chart of exosomes extraction. (B) Representative TEM photograph of exosomes (scale bar, 200nm). (C) Exosome concentration and size distribution were measured by nanoparticle tracking analysis (NTA) from three preparations. (D) Detection of exosome markers CD9 and flotillin 1 by immunoblotting. WT, wide type; NC, control.

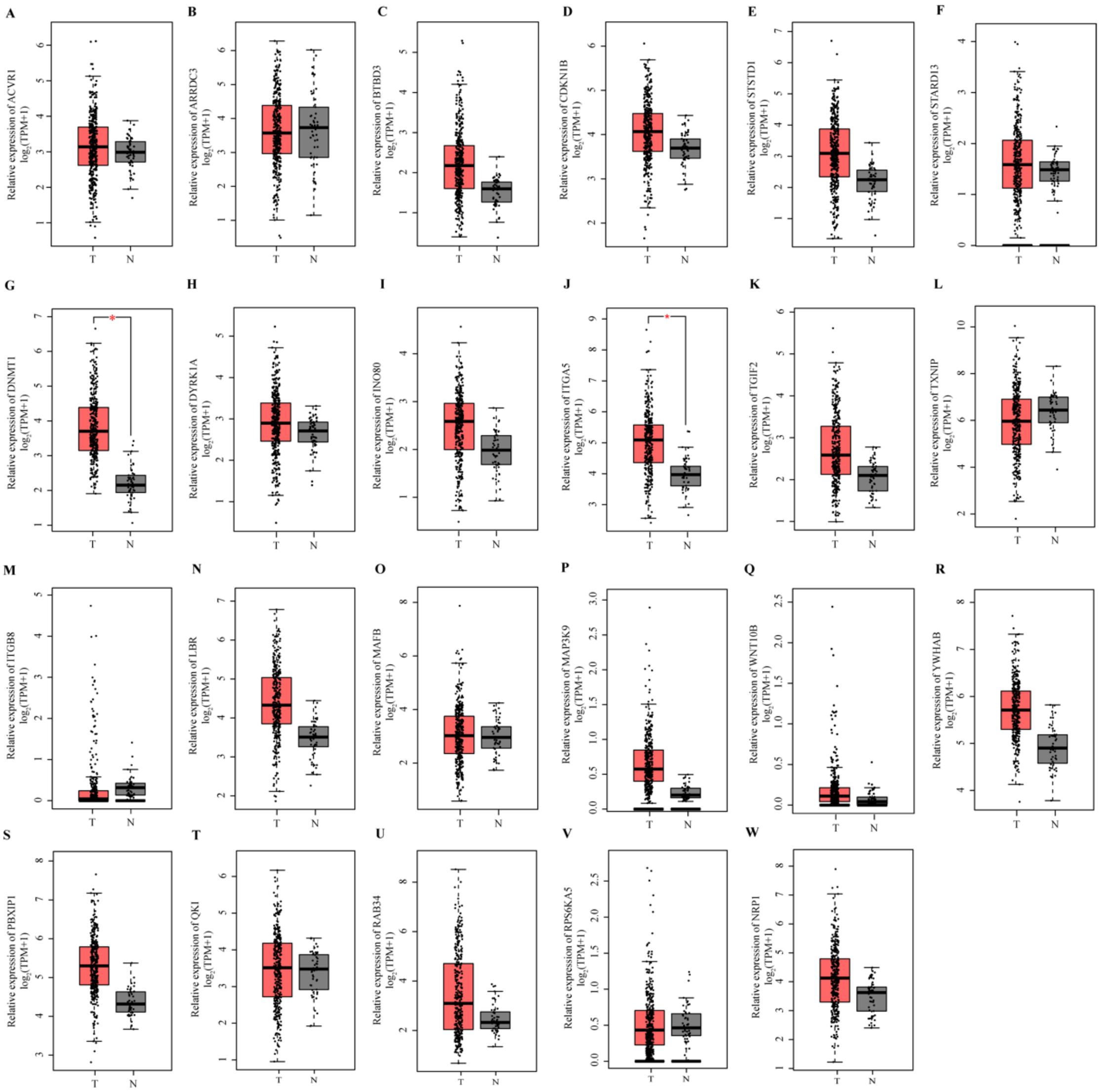

Figure S3. The expression of target genes of miR-148a-3p in the TCGA-LIHC database. *P<0.05.

Position 187-193 of ITGA5 3' UTR

```
ITGA5-3'UTR-WT  5'  ...GAUCCCUCCCCCCCAUGCACUGU...  3'
                                       |||||||
hsa-miR-148a-3p  3'    UGUUUCAAGACAUCACGUGACU      5'
ITGA5-3'UTR-MUT  5'  ...GAUCCCUCCCCCCCAACGUGACU...  3'
```

Position 902-909 of ITGA5 3' UTR

```
ITGA5-3'UTR-WT  5'... GUUCUGCCUGCCAGCUGCACUGA...  3'
                                      ||||||||
hsa-miR-148a-3p  3'    UGUUUCAAGACAUCACGUGACU      5'
ITGA5-3'UTR-MUT  5'... GUUCUGCCUGCCAGCACGUGACU...  3'
```

Figure S4. Sequencing maps between ITGA5'-3'UTR-WT and ITGA5'-3'UTR-MUT.
